# Analysis of glycaemic control with a connected smart pen cap in adults with type 1 diabetes: a randomised, open-label, parallel-group trial

**DOI:** 10.1007/s00125-026-06674-w

**Published:** 2026-02-03

**Authors:** Fernando Sebastian-Valles, Carolina Sager-La Ganga, Alicia Justel Enriquez, Sara Jimenez Blanco, Víctor Navas-Moreno, Jose Alfonso Arranz Martin, Mónica Marazuela

**Affiliations:** Department of Endocrinology and Nutrition, Hospital Universitario de La Princesa, Instituto de Investigación Sanitaria de La Princesa, Universidad Autónoma de Madrid, Madrid, Spain

**Keywords:** Digital health, Intention-to-treat analysis, Randomised clinical trial, Smart cap, Type 1 diabetes

## Abstract

**Aims/hypothesis:**

Connected insulin pens and smart caps automatically capture dosing data and can prompt patients about missed prandial boluses, but independent randomised evidence in type 1 diabetes is scarce. We aimed to assess whether a connected insulin pen cap improves glycaemic management compared with an otherwise identical non-connected cap.

**Methods:**

We conducted an investigator-initiated, randomised, open-label, parallel-group trial in a European public hospital over 8 weeks. Adults with type 1 diabetes treated with multiple daily injections and who had suboptimal glycaemic management (baseline time above range [>10.0 mmol/l, >180 mg/dl] >25%) were randomly assigned (1:1) to a connected pen cap (Insulclock 2.0; Insulcloud, Madrid, Spain) or an identical cap for which the Bluetooth connectivity had been disabled (disconnected). Allocation was performed using opaque sealed envelopes, and no masking was applied. The primary outcome was time above range, with two thresholds (>10.0 mmol/l, >180 mg/dl and >13.9 mmol/l, >250 mg/dl) based on intermittently scanned CGM downloads. Analyses followed the intention-to-treat principle, using longitudinal mixed models and multiple imputation. Secondary outcomes included time in range, glycaemic variability (standard deviation and coefficient of variation), HbA_1c_ and patient-reported outcomes (fear of hypoglycaemia).

**Results:**

Forty-two participants were randomised for inclusion in the study (21 per group: 25 women, 17 men). One withdrew before baseline, leaving 41 in the intention-to-treat analysis. Compared with the control group, the connected-cap group had lower time above range >13.9 mmol/l (mean difference −4.8 percentage points; 95% CI −9.5, −0.1; *p*=0.045) and reduced glucose standard deviation (−0.35 mmol/l; 95% CI −0.64, −0.06; *p*=0.018). HbA_1c_ showed a borderline reduction of −3.5 mmol/mol (−0.32%) (95% CI −7.1, 0.0 mmol/mol; −0.65, 0.00%; *p*=0.050). Per-protocol analyses suggested an 8% absolute increase in time in range among adherent users. No adverse events or device-related serious adverse events occurred. No between-group differences were observed in avoidance behaviours based on the hypoglycaemia fear survey significantly decreased in the treatment group (β=−2.44; 95% CI −4.45, −0.43; *p*=0.019).

**Conclusions/interpretation:**

In routine clinical care, use of a connected pen cap reduced severe hyperglycaemia, glycaemic variability and hypoglycaemia avoidance behaviours in adults with type 1 diabetes and suboptimal glycaemic management. These findings support the integration of low-burden, data-driven tools into public diabetes care. Larger and longer trials should evaluate the durability and cost-effectiveness of these interventions.

**Trial registration:**

ClinicalTrials.gov NCT06845891

**Funding:**

Investigator-initiated study without industry funding; devices were procured by the hospital. Funders had no role in study design, conduct, analysis or reporting

**Graphical Abstract:**

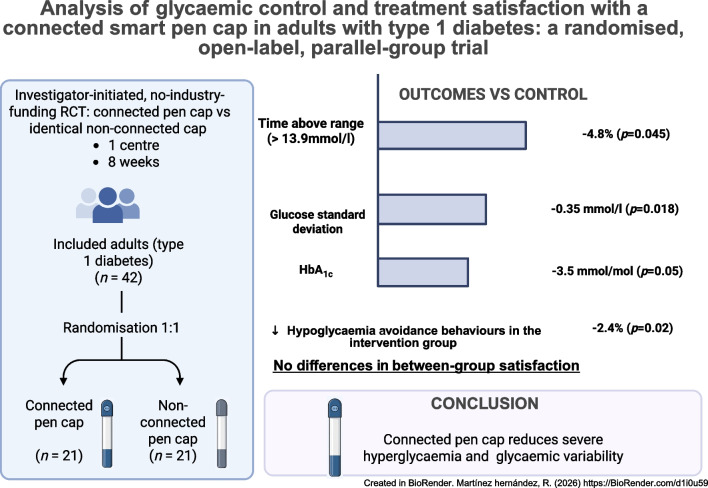

**Supplementary Information:**

The online version contains peer-reviewed but unedited supplementary material available at 10.1007/s00125-026-06674-w.


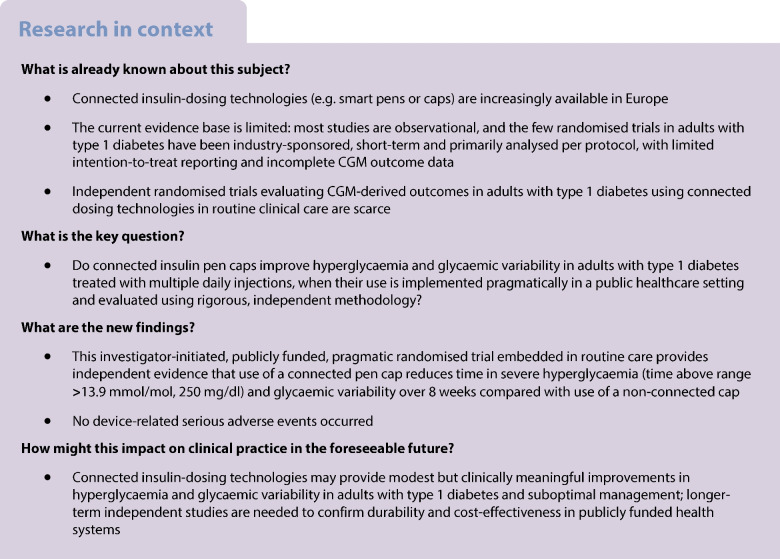


## Introduction

Sustained glycaemic management has been the cornerstone of type 1 diabetes management ever since pivotal trials demonstrated significant reductions in complications through use of intensive glucose targets [1]. However, achieving and maintaining glycaemic goals remains challenging in clinical practice, even more so than other metabolic targets such as blood pressure or lipid levels [2]. Among individuals treated with insulin, missed or delayed prandial boluses, dosing errors and barriers to timely dose adjustment are critical determinants of suboptimal glycaemic management, and have been associated with poorer clinical outcomes [3, 4]. Person-centred care and the use of objective and timely digital data are therefore key elements to support clinical decision-making and self-management.

Within this context, digital health technologies have evolved to integrate data from multiple sources (e.g. glucose sensors, physical activity, comorbidities). However, for many years, a standardised mechanism for the automatic capture of insulin-dosing data and their integration with other clinical information was lacking. Connected insulin pens and smart pen caps address this gap by recording and transmitting the timing and amount of insulin administered, providing reminders, and generating reports that can be reviewed during clinical consultations [5–8]. The currently available evidence, recently synthesised in a methodologically rigorous systematic review [9], indicates that this field remains at an early stage: most studies are observational with a small sample size, and only two randomised controlled trials in type 1 diabetes populations were identified [10, 11]. Nevertheless, the available studies consistently suggest potential improvements in glycaemic metrics, high patient satisfaction and indications of cost-effectiveness through use of such devices.

Critical gaps remain: (1) a lack of investigator-initiated randomised controlled trials independent of industry funding, which are essential to minimise sponsorship bias and enhance transparency and reproducibility; (2) the need for robust analytical methods (e.g. intention-to-treat analyses, longitudinal models, rigorous handling of missing data) applied in real-world clinical settings; and (3) the generation of evidence relevant to European public health systems, where adoption and reimbursement decisions rely on unbiased and generalisable data. Comparison with industry-sponsored trials is particularly important, as such studies frequently exhibit methodological limitations, including the absence of intention-to-treat analyses and incomplete reporting of key glycaemic metrics [12–14]. Furthermore, in this context, equitable allocation strategies are needed to avoid selection biases in real-world trials resulting from Hart’s inverse care law [15], which tends to favour individuals with better baseline glycaemic management.

To address these gaps, we conducted a parallel-group, open-label, investigator-initiated randomised clinical trial, independent of industry funding, comparing use of a connected insulin pen cap for prandial insulin with use of an identical non-connected cap, and integrated within routine care in a European public hospital. Our aim was to generate independent, rigorous and clinically actionable evidence to inform the integration of digital health tools into clinical practice and health policy decision-making across European healthcare systems.

## Methods

### Trial design

This was a randomised, parallel-group, open-label trial comparing the complete Insulclock 2.0 system (Insulcloud, Madrid, Spain), which comprises a connected insulin pen cap and its companion smartphone application that records and integrates insulin dosing, reminders and continuous glucose monitoring (CGM) data, against an identical cap in which the Bluetooth connectivity was technically disabled (disconnected). Adults were assigned 1:1 to the treatment or control groups. The trial was registered at ClinicalTrials.gov (NCT06845891) and approved by the research ethics committee of the Hospital Universitario de La Princesa, Madrid (Spain) before initiation (16 Jan 2025; CEIm 5350-02/2025). Screening, recruitment and follow-up occurred from 26 Feb 2025 to 19 June 2025. Written informed consent was obtained from all participants. The trial adhered to the terms of the Declaration of Helsinki and CONSORT 2025 guidelines [16].

### Participants: inclusion and exclusion criteria

Eligible participants were adults (≥18 years) with established type 1 diabetes treated with multiple daily insulin doses who were regular users of flash glucose monitoring with >70% data capture in recent downloads [17]. Additional inclusion criteria were adequate treatment adherence (no missed clinic appointments in the prior 12 months), baseline time above range (TAR: mean glucose >10.0 mmol/l [>180 mg/dl]) >25%, the ability to provide written informed consent, and access to a compatible smartphone with internet connectivity to allow follow-up via the Insulcloud application.

Exclusion criteria were current pregnancy, acute severe illness, contraindications to use of real-time CGM according to the Framework Agreement of the Community of Madrid, or the inability to provide informed consent or to comply with study procedures for any reason. The source population comprised adults with type 1 diabetes receiving routine care at a single tertiary public hospital in Madrid, Spain, within a universal healthcare system that provides full coverage of diabetes technologies. The study sample was approximately comparable to adults with long-standing type 1 diabetes treated with multiple daily injections in this setting in terms of age and gender distribution. Data on race or ethnicity were not collected, as these variables are not routinely recorded in clinical practice in Spain and were not required by the ethics committee. Information on individual socioeconomic status was not available; however, all participants were managed within the same publicly funded healthcare system. Sex/gender was self-reported by participants at enrolment.

### Setting and data collection

**Fig. 1:**
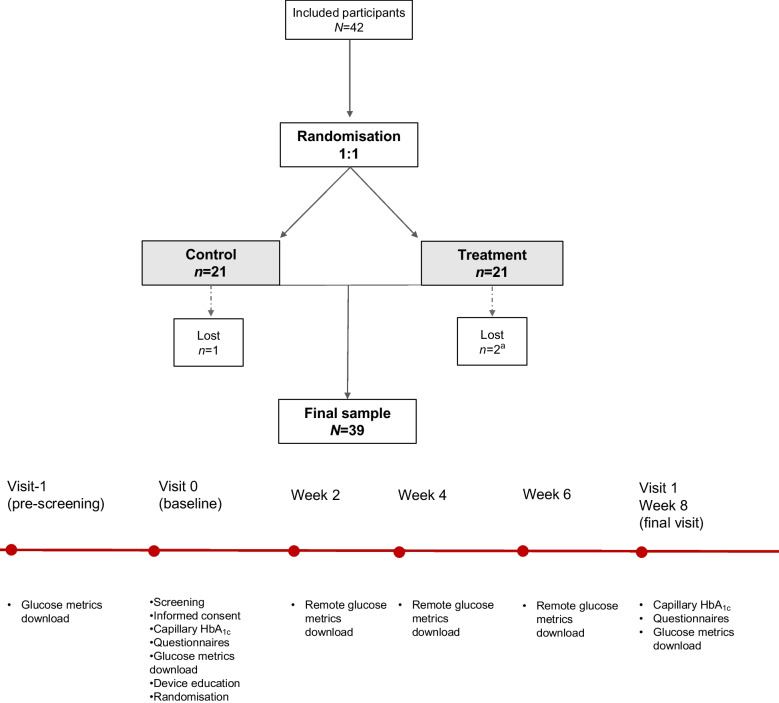
Flow diagram and study timeline. Flow of participants through the trial and schedule of assessments. A total of 42 participants were randomised 1:1 to the control or treatment group. Three participants did not complete the study (two in the treatment group and one in the control group). ^a^One of these participants in the treatment group withdrew during the baseline visit and had no available baseline or follow-up data

Consecutive participants were pre-screened (visit −1: CGM review) during routine endocrinology consultations at the Hospital Universitario de La Princesa. CGM data were obtained from the LibreView platform (Abbott), which was used independently of the Insulcloud application to download and manage intermittently scanned CGM data. After confirming pre-screening visit, participants were scheduled in paired groups (up to six participants per group) for a screening visit within 2–4 weeks. At the screening visit (visit 0: screening, baseline and randomisation), eligibility criteria were verified, study information was provided to the participants, and CGM data from the preceding 2 weeks were downloaded to confirm that participants met the glycaemic inclusion thresholds. Data were collected on paper case report forms, and entered into a password-protected anonymised electronic database stored on hospital computers. The study comprised three in-person visits: the pre-screening visit (visit −1), the baseline visit (visit 0) and a final visit at week 8 (visit 1: capillary HbA_1c_, questionnaires and last glucose metrics download). Between these visits, glucose metrics were downloaded remotely from the LibreView cloud every 14 days. Each participant therefore had five sensor data downloads in total (one at baseline and four during the intervention). The study flow and timeline are shown in Fig. 1.

At the baseline visit, participants provided written informed consent, underwent medical history verification and completed the baseline two-week sensor data download (LibreView). They also completed patient-reported questionnaires: the International Physical Activity Questionnaire [18] and the Spanish version of the Hypoglycaemia Fear Survey [19]. In addition, a capillary HbA_1c_ measurement was obtained. Basal insulin formulations included insulin degludec (Tresiba; Novo Nordisk), insulin glargine U100 (Lantus; Sanofi) and insulin glargine U300 (Toujeo; Sanofi). Prandial insulin analogues included insulin aspart (NovoRapid; Novo Nordisk), faster-acting insulin aspart (Fiasp; Novo Nordisk) and insulin lispro (Humalog; Eli Lilly). The basal:prandial insulin ratio was calculated as the proportion of the total daily insulin dose administered as basal insulin. The device-training session and randomisation took place at the same visit. The final visit (week 8) included verification of concomitant medications, repeat capillary HbA_1c_, repeat questionnaires and activation of the connected device for participants originally allocated to the control group. Adverse events and serious adverse events were prospectively recorded at the final study visit and through spontaneous reporting during the 8-week follow-up.

### Interventions

Fifty Insulclock 2.0 connected caps were acquired by the hospital for routine clinical use in the endocrinology outpatient clinic. In routine practice, allocation of technological resources is decided individually by the treating physician; however, to minimise the likelihood of Hart’s inverse care law [15], whereby those with greater healthcare needs are less likely to receive innovative interventions, to avoid preferential allocation to individuals with better baseline glycaemic management for the purposes of this study, and to evaluate the device under real-world clinical conditions, participants meeting inclusion criteria were randomised to receive either the connected cap (treatment group) or an identical cap without active connectivity (control group). Participants allocated to the control group were informed that their present cap could be activated free of charge after completion of the 8-week follow-up. Placement of the cap and instruction on device use were provided during the initial training session.

The Insulclock 2.0 system consists of a small cap that attaches to pens used for the administration of rapid-acting insulin analogues, with a companion smartphone application (Insulcloud). The device automatically records each injection, including time and dose, transmits these data to the app, and provides configurable reminders for missed or delayed boluses. The platform integrates insulin-dosing information with glucose data, and generates summary reports and visualisations, with the aim of improving adherence and supporting real-time treatment decisions.

### Prespecified outcomes

The primary outcome was the TAR, expressed as percentage time with glucose >10.0 mmol/l (>180 mg/dl) or >13.9 mmol/l (>250 mg/dl). Secondary outcomes included other sensor-derived glycaemic metrics: time in range (TIR: 3.9–10.0 mmol/l [70–180 mg/dl]) time in tight range (TITR: 3.9–7.8 mmol/l [70–140 mg/dl]), time below range (<3.9 mmol/l [<70 mg/dl] or <3.0 mmol/l [<54 mg/dl]), coefficient of variation, glucose standard deviation and the glucose management indicator. At the baseline and final visits, capillary HbA_1c_ was measured using an Afinion analyser (Abbott), and patient-reported outcomes were assessed using the questionnaires described above.

### Changes to outcomes after trial commencement

The original protocol defined the primary outcome as the TAR >10.0 mmol/l (>180 mg/dl) without sub-classification. Because the eligibility criteria included a baseline TAR >10.0 mmol/l (>180 mg/dl) >25%, the TAR >10.0 mmol/l (>180 mg/dl) values were high and relatively homogeneous between the groups at baseline, reducing the discriminatory capacity of this metric. Therefore, before unblinding and according to the statistical analysis plan, we prespecified reporting of TAR in two bands (10.0–13.9 mmol/l [180–250 mg/dl] and >13.9 mmol/l [>250 mg/dl]) to better capture clinically meaningful reductions in severe hyperglycaemia in this population, consistent with international consensus recommendations [17].

### Sample size calculation

Sample size was calculated allow detection of a reduction in TAR from a baseline mean of 40% (SD 15) to 25% (absolute difference 15 percentage points), with 80% power (β=0.20) and a two-sided α of 0.05. Under these assumptions, 34 participants were required. Allowing for an anticipated dropout rate of 20–30%, consistent with prior device studies, the target sample size was set at 42 participants.

### Randomisation, concealment and masking

The allocation sequence (1:1) was generated by randomly assigning treatment groups to sequential study numbers. Each allocation was placed into an opaque, sealed envelope. At the baseline visit, after providing written informed consent and completing assessments, participants were invited to select one envelope at random from the set prepared in advance. The content of the envelope determined their group assignment. Investigators were not involved in the selection process, and envelopes were identical and opaque, ensuring allocation concealment until assignment. The trial was open label; participants and treating clinicians were not masked to group allocation. Glycaemic outcomes were automatically obtained from LibreView downloads, and analyses were prespecified in the statistical analysis plan.

### Statistical analysis

Data for continuous variables are presented as median (IQR) and those for categorical variables are presented as *n* (%). Normality was assessed using both a statistical method (Kolmogorov–Smirnov test) and a graphical method (normal probability plots). Analyses were performed on an intention-to-treat (ITT) basis. Variables with markedly skewed distributions were log-transformed as appropriate.

For outcomes with two time points (baseline and end of study), we used ANCOVA, adjusting for the baseline value. Missing data were handled using multiple imputation by chained equations (MICE) with 20 imputations under a missing-at-random assumption; estimates were pooled using Rubin’s rules. Sensitivity analyses included complete-case analyses (without imputation) and per-protocol analyses, excluding participants lost to follow-up.

For outcomes with more than two repeated measurements (e.g. the five sequential sensor downloads), linear mixed-effects models with random intercepts for participants were used to account for within-participant correlation and longitudinal variability. This approach is recommended for longitudinal evaluations in clinical trials, as it provides less biased and more efficient estimates than simple before vs after or mean-change comparisons [20]. Multivariable models included baseline covariates exhibiting a standardised mean difference >0.20 between groups after randomisation, in line with recommended practice for assessing covariate balance in randomised trials [21]. In addition, outcomes were analysed using unadjusted simple linear regression models applied to longitudinal data, in which each participant contributed repeated measurements (five time points) for each glucose metric across the study period. All statistical analyses were performed using Stata version 17 (StataCorp) and JASP version 0.18.1 (JASP Team, University of Amsterdam, the Netherlands; available from https://jasp-stats.org [accessed 15 Jul 2025]). Two-sided *p* values <0.05 were considered statistically significant.

## Results

A total of 42 participants were randomised, with 21 allocated to each study group (Fig. 1). Three participants did not complete the study: two in the treatment group and one in the control group. One participant withdrew after signing the informed consent during the baseline visit, and had no available baseline or follow-up data. The remaining two participants completed baseline assessments and contributed CGM data during follow-up but did not use the study device or attend the final visit. The reasons for withdrawal were difficulty linking the CGM device to the monitoring platform and limited availability for follow-up visits (treatment group) and extended travel abroad during the study period preventing final evaluation (control group). Adverse events and serious adverse events were prospectively recorded; none were reported in either group during follow-up, and no device-related serious adverse events occurred.

### Baseline characteristics

The median age of the participants was 51.4 years (IQR 39.0–57.9); 59.5% were women, and the median diabetes duration was 21.7 years (IQR 7.7–34.7). The median baseline HbA_1c_ was 60 mmol/mol (IQR 53–68) (median 7.7%; IQR 7.0–8.4), with a median TIR of 58% (IQR 45–67). Baseline characteristics were well balanced between the groups (Table 1). Screening glucose metrics for patient selection are shown in electronic supplementary material (ESM) Table 1.

**Table 1:** Baseline characteristics of the study population

Variable	All patients	Control group	Treatment group
Age (years)	51.4 (39.0–57.9)	49.1 (42.6–56.1)	52.8 (39.0–58.5)
Gender (female) (%)^a^	25 (59.5)	14 (66.7)	11 (52.4)
Duration of diabetes (years)^a^	21.7 (7.7–34.7)	24.8 (15.7–34.7)	17.9 (5.9–36.7)
Current smoking^b^	11 (26.8)	6 (28.6)	5 (25.0)
BMI (kg/m^2^)^b^	26.1 (22.9–29.0)	25.8 (23.5–27.5)	27.7 (22.3–29.2)
Insulin dose (U/kg/day)^b^	0.64 (0.49–0.69)	0.61 (0.49–0.69)	0.64 (0.50–0.71)
Basal insulin (%)^b^			
Degludec	23 (56.1)	14 (66.7)	9 (45.0)
Glargine U300	16 (39.0)	6 (28.6)	10 (50.0)
Glargine U100	2 (4.9)	1 (4.8)	1 (5.0)
Basal insulin ratio (%)^b^	53 (48–67)	50 (48–67)	58 (51–66)
Prandial insulin (%)^b^			
Faster Aspart	15 (36.6)	9 (42.9)	6 (30.0)
Aspart	13 (31.7)	5 (23.8)	8 (40.0)
Lispro	13 (31.7)	7 (33.3)	6 (30.0)
Prandial insulin ratio (%)^b^	46 (33–51)	49 (33–52)	41 (33–49)
HbA_1c_ (mmol/mol)^b^	60 (53–68)	64 (53–69)	59 (53–64)
HbA_1c_ (%)^b^	7.7 (7.0–8.4)	8.0 (7.0–8.5)	7.6 (7.0–8.1)
METs (IPAQ)^b^	1746 (693–2772)	1746 (693–2946)	1593 (866–2644)
Diabetic retinopathy^a,b^	14 (34.1)	9 (42.9)	5 (25.0)
TIR (3.9–10.0 mmol/l) (%)	58 (45–67)	58 (44–69)	59 (46–66)
TITR(3.9–7.8 mmol/l) (%)	34 (20–44)	34 (23–42)	37 (19–45)
TAR >10.0 but <13.9 mmol/l (%)	24 (21–29)	26 (20–27)	24 (21–31)
TAR >13.9 mmol/l	11 (6–22)	13 (5–23)	11 (7.5–14)
TBR <3.9 but >3.0 mmol/l	1 (1–5)	2 (1–6)	1 (1–5)
TBR <3.0 mmol/l	0 (0–1)	0 (0–1)	0 (0–1)
Coefficient of variation (%)	37.1 (32.6–41.6)	37 (34–40.5)	38 (30.5–43.3)
Standard deviation (mmol/l)	3.5 (2.9–4.3)	3.5 (2.9–4.4)	3.5 (3.0–4.2)
GMI (%)	7.3 (6.9–7.8)	7.3 (6.9–7.8)	7.3 (7.0–7.8)
Number of hypoglycaemia events	4 (1–12)	4 (3–12)	5 (1–14)
Time in hypoglycaemia (min)	78 (45–105)	78 (45–98)	79 (40–122)
Perceived hypoglycaemia (HFS)^b^	1 (0–3)	1 (0–2)	1.5 (1–3)
HFS worry score^b^	41 (35–57)	38 (35–56)	45 (35–57)
HFS avoidance score^b^	16 (12–18)	16 (15–19)	14 (11–16)
HFS hyperglycaemia score^b^	7 (5–9)	7 (4–9)	7 (5.5–9)

Data are medians (IQR) for continuous variables and *n* (%) for categorical variables

^a^Variables with baseline imbalance (standardised mean difference >0.20); these are included as covariates in adjusted models

^b^Data for these variables were only available for 41 participants; baseline CGM data were available for all 42 participants

GMI, glucose management indicator; TBR, time below range; HFS, Hypoglycaemia Fear Survey [21]; IPAQ, International Physical Activity Questionnaire [20]

### Primary outcome: TAR

**Fig. 2:**
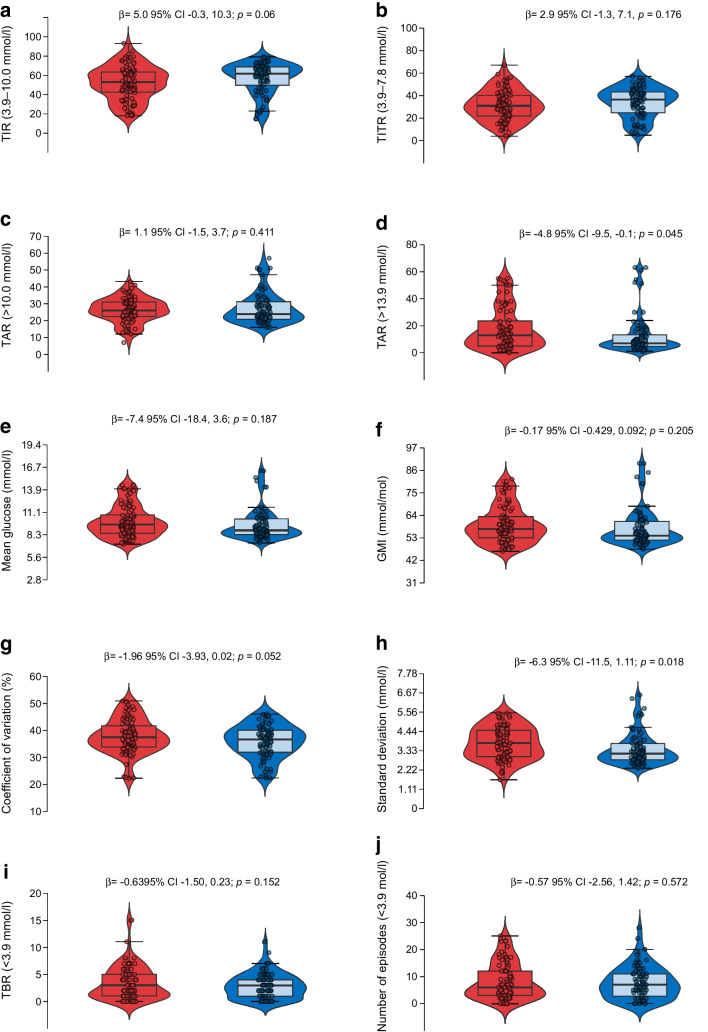
Unadjusted between-group comparisons of glycaemic metrics across the 8-week follow-up (ITT analysis). Red: control group; blue: treatment group. The violin plots illustrate the distribution of sensor-derived glycaemic metrics across all post-baseline downloads (four per participant) during the 8-week follow-up. Baseline values prior to treatment initiation were excluded. Metrics include TIR (3.9–10.0 mmol/l [70–180 mg/dl]), TITR (3.9–7.8 mmol/l [70–140 mg/dl]), TAR (>10.0 mmol/l [>180 mg/dl] and >13.9 mmol/l [>250 mg/dl), mean glucose, glucose management indicator (GMI), coefficient of variation, glucose standard deviation, time below range (TBR: <3.9 mmol/l [<70 mg/dl]) and number of hypoglycaemic episodes (<3.9 mmol/l [<70 mg/dl]). Within each violin plot, the embedded box-and-whisker plot shows the median (horizontal line) and IQR (box), with whiskers extending to the most extreme values within 1.5 × IQR. Individual observations are shown as jittered points. *p* values and 95% CIs are derived from unadjusted linear regression models (ITT)

At 8 weeks, the treatment group showed lower median TAR values compared with the control group: 23% (IQR 20–29) vs 27% (IQR 21–31 for glucose >10.0 mmol/l (>180 mg/dl) and 6% (IQR 4–12) vs 13% (IQR 6–20) for glucose >13.9 mmol/l (>250 mg/dl). In unadjusted longitudinal analyses of the four post-baseline sensor downloads (ITT analyses), the treatment group experienced a significant reduction in TAR >13.9 mmol/l (>250 mg/dl) compared with the control group (mean difference −4.8%; 95% CI −9.5, −0.1; *p*=0.045) (Fig. 2).

**Fig. 3:**
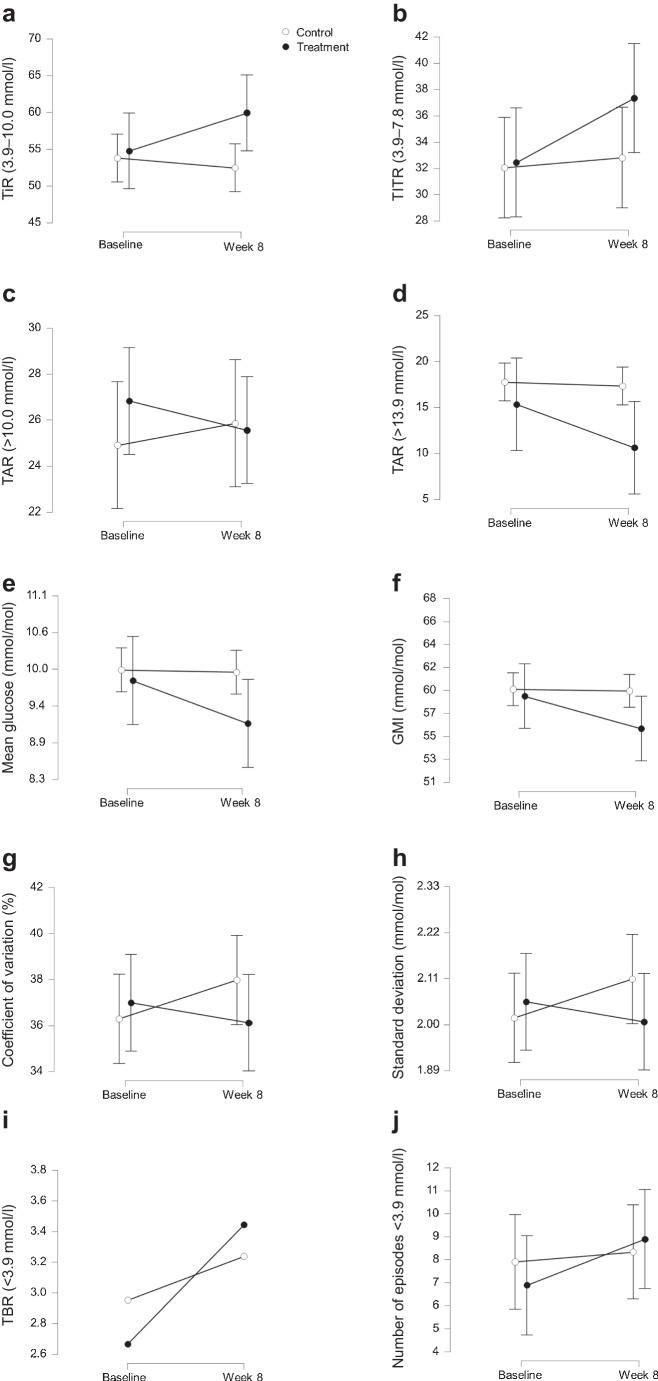
Unadjusted descriptive changes in CGM-derived glycaemic metrics from baseline to week 8 in the ITT population. Data represent unadjusted median values (IQR) for CGM-derived glycaemic metrics at baseline and at week 8 for the control group (open circles) and the treatment group (filled circles). Error bars represent the IQR. Statistical inference was based on linear mixed-effects models, not on these unadjusted descriptive comparisons

Figure 3 presents a descriptive visual analysis of the ITT population, aimed at providing an intuitive overview of the temporal evolution of CGM-derived metrics. This unadjusted visualisation is intended solely to aid interpretation of treatment trends; all statistical inferences and adjusted effect estimates were derived from linear mixed-effects models accounting for covariates and repeated measures.

**Fig. 4:**
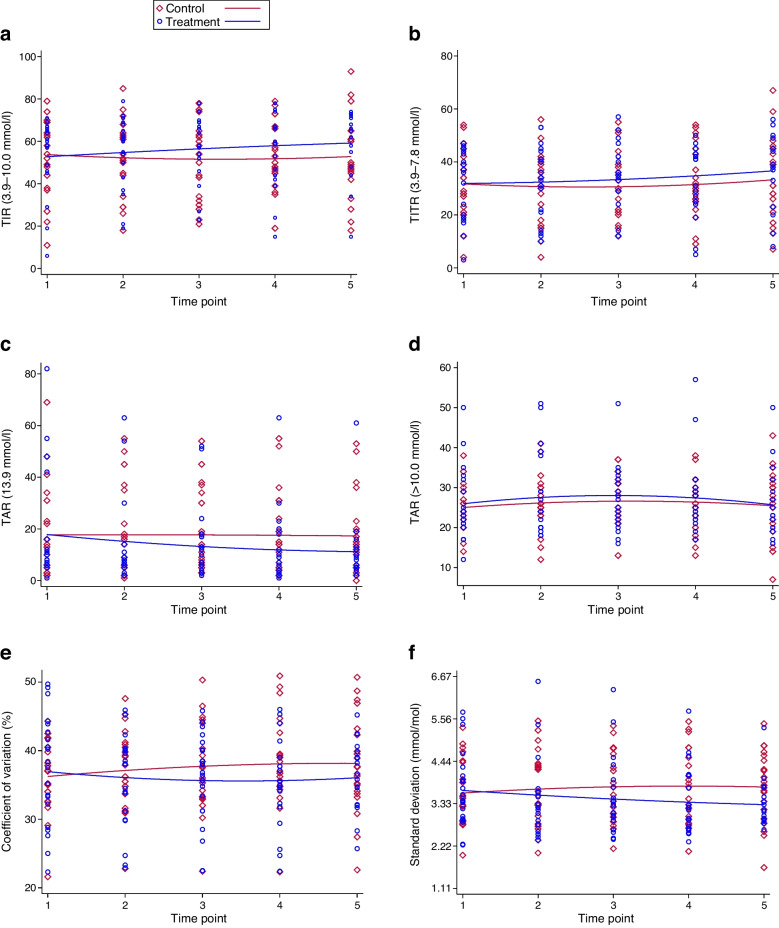
Longitudinal distribution of sensor-derived glycaemic metrics throughout the 8-week trial. Scatter plots showing the longitudinal trajectories of sensor-derived glycaemic metrics in the control group (red lines and symbols) and the treatment group (blue lines and symbols) across the five study time points (baseline and four post-baseline downloads). Metrics include TIR (3.9–10.0 mmol/l [70–180 mg/dl]), TITR (3.9–7.8 mmol/l [70–140 mg/dl]), TAR (>10.0 mmol/l (>180 mg/dl] and >13.9 mmol/l [>250 mg/dl]), coefficient of variation and glucose standard deviation. Lines represent fitted values from unadjusted linear regression models over time (ITT analysis)

In linear mixed-effects models adjusted for gender, diabetes duration and diabetic retinopathy (covariates that were selected because they showed baseline standardised mean differences >0.20 between groups; ESM Table 2), treatment allocation significantly modified the longitudinal trajectory of log-transformed TAR >13.9 mmol/l (>250 mg/dl) (β=−0.12; 95% CI −0.23, −0.01; *p*=0.034). Longer diabetes duration was independently associated with a higher TAR value for the >13.9 mmol/l (>250 mg/dl) threshold (β=0.033; 95% CI 0.016, 0.049; *p*<0.001), but gender and diabetic retinopathy were not significantly associated with TAR >13.9 mmol/l (>250 mg/dl) (Fig. 4).

### Secondary outcomes: glycaemic metrics

At 8 weeks, participants in the treatment group exhibited a lower median of the individual mean glucose values of 8.8 mmol/l (IQR 8.3–9.2) compared with 9.9 mmol/l (IQR 8.5–10.7) in the control group, and a higher TIR (65.5% [IQR 55–72] vs 49% [IQR 45–61]) and TITR (38% [IQR 33–49] vs 31% [IQR 23–43]) and a lower glucose standard deviation (3.1 mmol/l [55.2 mg/dl] vs 3.8 mmol/l [68.6 mg/dl]) compared with the control group.

In unadjusted longitudinal analyses, the treatment group demonstrated a significant reduction in glucose standard deviation compared with the control group (mean difference −0.35 mmol/l; 95% CI −0.64, −0.06; *p*=0.018) and a trend toward improved TIR and coefficient of variation that did not reach statistical significance (Figs. 2 and 3).

In adjusted mixed-effects models, treatment allocation significantly influenced the longitudinal trajectory of log-transformed glucose standard deviation (β=−0.024; 95% CI −0.046, −0.001; *p*=0.042). Longer diabetes duration was associated with higher glycaemic variability (β=0.008; 95% CI 0.003, 0.014; *p*=0.004). The likelihood-ratio test confirmed that inclusion of random intercepts significantly improved model fit (χ^2^=153.8, *p*<0.001). To complement these longitudinal analyses, Table 2 summarises the descriptive post-baseline all glycaemic metrics for both study groups, providing a clear comparison of unadjusted medians and IQRs, together with linear mixed-effects model- and ANCOVA-derived *p* values adjusted for gender, diabetes duration and diabetic retinopathy.

**Table 2:** Descriptive statistics for all CGM-derived metrics across all post-baseline visits (time points 2–5) for the control and treatment groups

Glycaemic metric	Control group (*n*=21)	Treatment group (*n*=20)	*p* value
TIR (3.9–10.0 mmol/l) (%)	53 (42–64)	61.5 (49–68.5)	0.109^a^
TITR (3.9–7.8 mmol/l) (%)	31 (22–40)	36.5 (24.5–43.5)	0.515^a^
Mean glucose (mmol/l)	9.6 (8.5–10.8)	8.9 (8.4–10.3)	0.274^a^
Standard deviation (mmol/l)	3.8 (3.0–4.5)	3.2 (2.8–3.7)	0.042^a^
Coefficient of variation (%)	37.5 (33.7–42.3)	36.5 (31.9–40.1)	0.057^a^
TAR >10.0 but <13.9 mmol/l (%)	26 (22–31)	24 (20.5–31.5)	0.539^a^
TAR >13.9 mmol/l	13 (5–24)	7 (4.5–13.5)	0.034^a^
TBR <3.9 but >3.0 mmol/l	3 (1–5)	3 (1–4)	0.945^a^
TBR <3.0 mmol/l	0 (0–1)	0 (0–0)	0.617^a^
Number of hypoglycaemic episodes	6 (3–12)	7 (2.5–11)	0.874^a^
GMI (%)	7.4 (7.0–8.0)	7.1 (6.9–7.8)	0.286^a^
HbA_1c_ (mmol/mol)^c^	62 (53–72)	56 (52–65)	0.050^b^
HbA_1c_ (%)^c^	7.8 (7.0–8.7)	7.3 (6.9–8.1)	0.050^b^
Insulin (U/kg/day)^c^	0.56 (0.47–0.64)	0.49 (0.36–0.72)	0.914^b^

Data are medians (IQR)

^a^Model adjusted for gender, diabetes duration and diabetic retinopathy

^b^*p* values derived from ANCOVA models adjusted for gender, diabetes duration and diabetic retinopathy

^c^HbA_1c_ and insulin dose refer to baseline measurements

GMI, glucose management indicator; TBR, time below range

Total daily insulin requirements showed a small but statistically significant reduction from baseline to week 8 in the overall cohort (mean difference −0.056 U/kg/day; 95% CI −0.102, −0.011; *p*=0.016). In exploratory within-group analyses, both the control and treatment groups showed modest decreases (control group: −0.054 U/kg/day, *p*=0.139; treatment group: −0.059 U/kg/day, *p*=0.047). However, in the adjusted ANCOVA model that included baseline insulin dose, treatment allocation, gender, diabetic retinopathy and diabetes duration, treatment group was not independently associated with insulin dose at week 8 (β=−0.005, *p*=0.914). Baseline insulin dose was the only significant predictor of final insulin requirements (β=0.83, *p*<0.001).

### Sensitivity analyses

Per-protocol analyses, which excluded the two participants lost to follow-up, confirmed improvements in TIR in the treatment group compared with the control group in the unadjusted analysis (median 62% [IQR 52–69] vs 54% [IQR 43–65], *p*=0.03). In multivariable adjusted models, the treatment group showed a significant increase in TIR (β=1.64; 95% CI 0.06, 3.22; *p*=0.041) and a reduction in glucose standard deviation (β=−1.85; 95% CI −3.43, −0.26; *p*=0.022) (ESM Table 3).

### Secondary outcomes: HbA_1c_ and patient-reported outcomes

ANCOVA models adjusting for gender, diabetes duration and diabetic retinopathy showed a borderline reduction in HbA_1c_ in the treatment group compared with the control group (β=−3.53 mmol/mol; 95% CI −7.07, 0.01; *p*=0.050) (Table 3), corresponding to an estimated difference of −0.32% (95% CI −0.65, 0.00%).

**Table 3:** ANCOVA model for change in HbA_1c_ at 8 weeks

Variable	Coefficient	95% CI	*p* value
HbA_1c_ at baseline (mmol/mol)	0.948	0.793, 1.103	<0.001
Treatment group^a^	−3.53	−7.07, 0.01	0.050
Diabetes duration (years)	0.007	−0.009, 0.023	0.370
Diabetic retinopathy (yes vs no)	−0.079	−0.530, 0.371	0.722
Gender (female vs male)	−0.210	−0.555, 0.136	0.225
Constant^b^	0.547	−0.614, 1.708	0.345

Results from an ANCOVA model assessing the effect of treatment allocation on HbA_1c_ at 8 weeks, adjusted for baseline HbA_1c_, expressed in mmol/mol (with % given in parentheses in the text), gender, diabetes duration and diabetic retinopathy

^a^The control group was used as the reference category

^b^Model intercept

Scores on the Hypoglycaemia Fear Survey showed a significant reduction in avoidance behaviours in the treatment group (β=−2.44; 95% CI −4.45, −0.43; *p*=0.019), with no significant differences in the worry or hyperglycaemia subscales (ESM Tables 4 and 5). Results without imputation are shown in ESM Table 6.

## Discussion

This randomised, investigator-initiated clinical trial conducted independently of industry funding provides the first evidence from a real-world, public hospital setting in Europe regarding the efficacy of use of a connected insulin pen cap for individuals with type 1 diabetes treated with multiple daily injections. Using ITT analyses with longitudinal models, the intervention was found to result in a significant reduction in time spent in severe hyperglycaemia **(**TAR >13.9 mmol/l [>250 mg/dl]), lower glucose variability and fewer hypoglycaemia avoidance behaviours, with a reduction in HbA_1c_ that did not reach conventional statistical significance after 8 weeks.

Poor adherence to prandial insulin administration is a well-recognised barrier to optimal glycaemic management. Up to 43% of individuals with type 1 diabetes miss at least one prandial bolus per week [3], and 20–45% inject at times different from those prescribed [4]. Common reasons include forgetfulness, fear of hypoglycaemia, dosing errors or accidental double administration [6]. These behaviours contribute to sustained hyperglycaemia [22], which is captured by TAR, a metric that is increasingly recognised as clinically meaningful [23, 24] and is associated with complications such as diabetic retinopathy, even among individuals with target HbA_1c_ levels [25].

The observed improvements in TAR and glucose standard deviation are clinically relevant. Glucose variability has been linked to inflammatory responses [26–28] and the development of microvascular complications [29], and represents a key therapeutic target beyond mean glucose [23, 25]. Reduction in hypoglycaemia avoidance behaviours, such as preventive under-dosing, frequent carbohydrate intake or exercise avoidance, suggests increased confidence in insulin administration and a more stable glycaemic profile. However, we did not collect data on treatment adherence in the control group, which limits direct comparisons of behavioural changes between groups.

Our findings align with previous observational studies and with the small number of randomised trials available to date evaluating connected insulin pens or smart caps (all of which have been industry-sponsored), which reported modest but meaningful improvements in glycaemic outcomes [8, 10, 11, 30]. However, those trials often lacked ITT analyses or complete reporting of glycaemic metrics, limiting their generalisability. By contrast, our investigator-led design, with transparent randomisation and rigorous analytical methods (including mixed-effects models, multiple imputation, and both ITT and per-protocol analyses) addresses some of these gaps and strengthens the reliability of the findings. Notably, the per-protocol analyses showed improvements in median TIR of 8% among adherent users, comparable to those of prior trials [10, 11], further supporting the consistency of the effect. This study also used a pragmatic allocation strategy that was designed to counter Hart’s inverse care law [15], whereby individuals with less optimal glycaemic management are often less likely to receive innovative technologies. By randomising eligible participants with suboptimal glycaemic management, we ensured equitable access to the intervention and generated evidence that is directly applicable to the populations who are most likely to benefit in routine clinical care.

Several limitations must be acknowledged. First, the sample size and study duration, although comparable to those of similar device studies [10, 11, 31, 32], precluded assessment of long-term or hard clinical outcomes, such as hospital admissions, acute metabolic complications or the development of chronic microvascular or macrovascular complications. Second, the open-label design may have introduced performance or observation bias [33]; however, glycaemic metrics were objectively downloaded from the LibreView cloud, reducing the risk of differential outcome ascertainment. Third, as a single-centre study conducted within a universal healthcare system with full reimbursement for diabetes technologies, external generalisability to other healthcare settings, particularly those with socioeconomic barriers to access, may be limited. Fourth, the inclusion criteria, requiring multiple daily insulin injections and a TAR >25% (>10.0 mmol/l [>180 mg/dl]), may limit the external validity of our findings to this specific clinical context. Fifth, although baseline covariates were balanced, residual confounding cannot be completely ruled out. Finally, although gender was included as a covariate in adjusted models, the trial was not powered for sex/gender-stratified analyses or treatment-by-sex/gender interaction testing; therefore, potential sex/gender-related heterogeneity cannot be excluded, and the generalisability of effect size across sexes/genders should be confirmed in larger studies.

Despite these limitations, this trial has important clinical and policy implications. It demonstrates that providing connected pen caps to individuals with suboptimal glycaemic management in real-world public healthcare settings can lead to measurable improvements in glycaemic management in type 1 diabetes. These findings support the integration of use of data-driven digital interventions into routine diabetes care, particularly in public health systems where equitable allocation is critical.

In conclusion, use of a connected insulin pen cap that automatically records the dose and timing of prandial insulin administration can improve key glycaemic outcomes and behavioural metrics in adults with type 1 diabetes and suboptimal glycaemic management. Investigator-initiated randomised trials are essential to build an independent evidence base for use of digital health tools, ensuring their appropriate adoption within healthcare systems.

## Supplementary Information

Below is the link to the electronic supplementary material.

ESM Tables (PDF 344 KB)

## Data Availability

Data sharing of de-identified participant data will be considered upon reasonable request after approval of a protocol and signing of a data transfer agreement with the corresponding author after publication of all secondary outcomes of this trial. Related documents are available from the corresponding author.

## Abbreviations

CGM: Continuous glucose monitoring
ITT: Intention to treat
TAR: Time above range
TIR: Time in range
TITR: Time in tight range
