# Correction: Analysis of glycaemic control with a connected smart pen cap in adults with type 1 diabetes: a randomised, open-label, parallel-group trial

**DOI:** 10.1007/s00125-026-06786-3

**Published:** 2026-07-02

**Authors:** Fernando Sebastian-Valles, Carolina Sager-La Ganga, Alicia Justel Enriquez, Sara Jimenez Blanco, Víctor Navas-Moreno, Jose Alfonso Arranz Martin, Mónica Marazuela

**Affiliations:** Department of Endocrinology and Nutrition, Hospital Universitario de La Princesa, Instituto de Investigación Sanitaria de La Princesa, Universidad Autónoma de Madrid, Madrid, Spain

**Correction: Diabetologia (2026) 69:1191-1204**

10.1007/s00125-026-06674-w

All mention of treatment satisfaction data has been removed from the original publication as the authors mistakenly used the Diabetes Treatment Satisfaction Questionnaire change version (DTSQc) rather than the status version (DTSQs) to collect baseline data. Consequently, baseline DTSQc scores and analyses adjusting for baseline DTSQc values can not be interpreted or relied upon as validated assessments of treatment satisfaction.

This issue does not affect the primary outcome, the CGM-derived glycaemic metrics, the HbA_1c_ analyses, the hypoglycaemia fear survey results or the principal conclusions of the study regarding glycaemic outcomes associated with use of the connected insulin pen cap.

The authors also acknowledge that the Spanish DTSQc translation used in this study was an unauthorised version that was not obtained from Health Psychology Research Ltd (HPR), the copyright holder of the DTSQ instruments (www.healthpsychologyresearch.com; info@healthpsychologyresearch.com).

The following references relating to the development and intended use of the DTSQs and DTSQc should also have been cited:

1. Bradley C, Lewis KS (1990) Measures of psychological well-being and treatment satisfaction developed from the responses of people with tablet-treated diabetes. Diabet Med 7:445–451
2. Bradley C (1994) The Diabetes Treatment Satisfaction Questionnaire: DTSQ. In: Bradley C, editor. Handbook of Psychology and Diabetes: A Guide to Psychological Measurement in Diabetes Research and Practice. Abingdon: Routledge, formerly Harwood Academic Publishers, pp 111–132
3. Howorka K, Pumprla J, Schlusche C, Wagner-Nosiska D, Schabmann A, Bradley C (2000) Dealing with ceiling baseline treatment satisfaction level in patients with diabetes under flexible, functional insulin treatment: assessment of improvements in treatment satisfaction with a new insulin analogue. Qual Life Res 9:915–930
4. Bradley C, Plowright R, Stewart J, Valentine J, Witthaus E (2007) The Diabetes Treatment Satisfaction Questionnaire change version (DTSQc) evaluated in insulin glargine trials shows greater responsiveness to improvements than the original DTSQ. Health Qual Life Outcomes 5:57

The authors apologise for the lack of clarity regarding administration and reporting of the DTSQc. All treatment satisfaction data has now been removed and the original article has been corrected.

