# Supplementary material for: Analysis of glycaemic control with a connected smart pen cap in adults with type 1 diabetes: a randomised, open-label, parallel-group trial
Source: Diabetologia. 2026 Feb 3;69(5):1191–204. doi: 10.1007/s00125-026-06674-w (PMC13005853; doi:10.1007/s00125-026-06674-w)
Supplement: Supplementary file 1 — ESM Tables (PDF 344 KB) [file 125_2026_6674_MOESM1_ESM.pdf]

# Electronic supplementary material Table 1

**Glucose Metrics at pre-screening visit (visit -1)**

| Variable | All patients<br>Median [IQR] | Control<br>Median [IQR] | Treatment<br>Median [IQR] | P value |
|---|---|---|---|---|
| TIR (%) | 54 [40–64] | 52 [41–63] | 60 [40–66] | 0.537 |
| TITR (%) | 33 [20–41] | 31 [20–39] | 37 [18–43] | 0.676 |
| TAR >10.0 mmol/l (%) | 25 [23–30] | 25 [23–28] | 26 [23–30] | 0.595 |
| TAR >13.9 mmol/l (%) | 8.5 [6–23] | 16 [7–23] | 8 [6–16] | 0.289 |
| TBR <3.9 mmol/l (%) | 2 [1–4] | 2 [1–5] | 2 [1–3] | 0.904 |
| TBR <3.0 mmol/l (%) | 0 [0–1] | 0 [0–1] | 0 [0–0] | 0.578 |
| CV (%) | 37 [32.8–40.6] | 37 [32.9–40.6] | 36.4 [31.7–40.5] | 0.694 |
| GMI (mmol/mol) | 57.4 [53.0–63.9] | 59.5 [54.1–63.9] | 57.4 [53.0–61.7] | 0.666 |
| Number of hypoglycaemia events | 5 [1–13] | 5 [1–13] | 5 [2–10] | 0.915 |
| Time in hypoglycaemia (min) | 75.5 [45–98] | 77 [45–93] | 74 [40–98] | 0.807 |

# Electronic supplementary material Table 2

**Supplementary Table. Standardized Mean Differences at Baseline**

| Variable | Control<br>Mean (SD) or % | Treatment<br>Mean (SD) or % | Standardized<br>Difference |
|---|---|---|---|
| Age (years) | 48.18 (13.71) | 48.90 (17.61) | -0.046 |
| Sex (female, %) | 66.7% | 52.4% | 0.287 |
| BMI (kg/m²) | 26.48 (5.13) | 26.41 (4.71) | 0.016 |
| Duration of diabetes (years) | 24.22 (12.09) | 20.35 (16.53) | 0.267 |
| $HbA_{1}c$ (mmol/mol) | 63.3 (12.0) | 61.0 (12.6) | 0.109 |
| HbA1c (%) | 7.94 (1.10) | 7.82 (1.15) | |
| Smoking habit (%) | 28.6% | 25.0% | 0.079 |
| Diabetic retinopathy (%) | 42.9% | 25.0% | 0.375 |
| Insulin (U/kg/day) | 0.61 (0.17) | 0.62 (0.16) | -0.047 |
| Physical activity (IPAQ METs) | 2299 (2012) | 2619 (3367) | -0.115 |
| GLP-1 agonist | 14.3% | 5% | 0.311 |
| SGLT-2 inhibitors | 4.8% | 5% | -0.011 |

# Electronic supplementary material Table 3

Per-protocol mixed-effects regression for TIR

| Variable | Coefficient | Std. Error | z | p-value | 95% CI Lower | 95% CI Upper |
|---|---|---|---|---|---|---|
| Moment 2 | 0.1225814 | 1.823704 | 0.07 | 0.946 | -3.451812 | 3.696975 |
| Moment 3 | -0.340426 | 1.95955 | -0.17 | 0.862 | -4.181074 | 3.500221 |
| Moment 4 | -1.695522 | 2.136246 | -0.79 | 0.427 | -5.882487 | 2.491443 |
| Moment 5 | -1.315948 | 2.364624 | -0.56 | 0.578 | -5.950527 | 3.31863 |
| Treatment arm (1=treatment) | -4.126384 | 5.353115 | -0.77 | 0.441 | -14.6183 | 6.365528 |
| Sex (1=male) | -6.14261 | 4.961925 | -1.24 | 0.216 | -15.86781 | 3.582585 |
| Diabetic retinopathy | 8.678516 | 6.521364 | 1.33 | 0.183 | -4.103123 | 21.46016 |
| Duration of diabetes | -0.4982372 | 0.2210185 | -2.25 | 0.024 | -0.9314255 | -0.0650488 |
| **Treatment arm × Moment** | **1.643122** | **0.8054317** | **2.04** | **0.041** | 0.0645052 | 3.221739 |
| Constant | 65.94295 | 6.1491 | 10.72 | 0.0 | 53.89094 | 77.99497 |

Per-protocol mixed-effects regression for TTIR

| Variable | Coefficient | Std. Error | z | p-value | 95% CI Lower | 95% CI Upper |
|---|---|---|---|---|---|---|
| Moment 2 | -0.5222129 | 1.651546 | -0.32 | 0.752 | -3.759183 | 2.714758 |
| Moment 3 | 0.3919194 | 1.774455 | 0.22 | 0.825 | -3.085949 | 3.869787 |
| Moment 4 | -0.5406052 | 1.934598 | -0.28 | 0.78 | -4.332348 | 3.251137 |
| Moment 5 | 1.234196 | 2.141416 | 0.58 | 0.564 | -2.962903 | 5.431295 |
| Treatment arm (1=treatment) | -2.098142 | 4.15825 | -0.5 | 0.614 | -10.24816 | 6.051878 |
| Sex (1=male) | -6.270667 | 3.677165 | -1.71 | 0.088 | -13.47778 | 0.9364442 |
| Diabetic retinopathy | 8.636212 | 4.831753 | 1.79 | 0.074 | -0.8338495 | 18.10627 |
| Duration of diabetes | -0.3362802 | 0.1637584 | -2.05 | 0.04 | -0.6572408 | -0.0153195 |
| **Treatment arm × Moment** | **0.8087528** | **0.7292806** | **1.11** | **0.267** | **-0.6206109** | **2.238117** |
| Constant | 40.37484 | 4.611081 | 8.76 | 0.0 | 31.33728 | 49.41239 |

Per-protocol mixed-effects regression for TAR> 13.0 mmol/mol

| Variable | Coefficient | Std. Error | z | p-value | 95% CI Lower | 95% CI Upper |
|---|---|---|---|---|---|---|
| Moment 2 | 1.923312 | 1.144188 | 1.68 | 0.093 | -0.3192562 | 4.16588 |
| Moment 3 | 1.956462 | 1.229287 | 1.59 | 0.111 | -0.4528965 | 4.365821 |
| Moment 4 | 1.386889 | 1.340293 | 1.03 | 0.301 | -1.240037 | 4.013815 |
| Moment 5 | 0.6932115 | 1.483576 | 0.47 | 0.64 | -2.214543 | 3.600966 |
| Treatment arm (1=treatment) | 1.970873 | 2.682386 | 0.73 | 0.462 | -3.286507 | 7.228254 |
| Sex (1=male) | 0.3088227 | 2.303073 | 0.13 | 0.893 | -4.205117 | 4.822763 |
| Diabetic retinopathy | -1.430978 | 3.02577 | -0.47 | 0.636 | -7.361378 | 4.499423 |
| Duration of diabetes | 0.0265214 | 0.1025511 | 0.26 | 0.796 | -0.1744751 | 0.2275179 |
| Treatment arm × Moment | -0.3689034 | 0.5051905 | -0.73 | 0.465 | -1.359059 | 0.6212519 |
| Constant | 24.36216 | 2.910734 | 8.37 | 0.0 | 18.65723 | 30.0671 |

Per-protocol mixed-effects regression for TAR> 13.9 mmol/mol

| Variable | Coefficient | Std. Error | z | p-value | 95% CI Lower | 95% CI Upper |
|---|---|---|---|---|---|---|
| Moment 2 | -2.338231 | 1.540565 | -1.52 | 0.129 | -5.357682 | 0.6812209 |
| Moment 3 | -1.400599 | 1.655376 | -0.85 | 0.398 | -4.645077 | 1.843879 |
| Moment 4 | -0.1199107 | 1.804576 | -0.07 | 0.947 | -3.656815 | 3.416994 |
| Moment 5 | -0.20916 | 1.997499 | -0.1 | 0.917 | -4.124186 | 3.705866 |
| Treatment arm (1=treatment) | 3.199441 | 5.10821 | 0.63 | 0.531 | -6.812467 | 13.21135 |
| Sex (1=male) | 6.797549 | 4.861356 | 1.4 | 0.162 | -2.730534 | 16.32563 |
| Diabetic retinopathy | -9.325085 | 6.38995 | -1.46 | 0.144 | -21.84916 | 3.198987 |
| Duration of diabetes | 0.4838669 | 0.2165623 | 2.23 | 0.025 | 0.0594127 | 0.9083211 |
| Treatment arm × Moment | -1.200474 | 0.6804433 | -1.76 | 0.078 | -2.534118 | 0.1331706 |
| Constant | 5.734745 | 5.986933 | 0.96 | 0.338 | -5.999428 | 17.46892 |

Per-protocol mixed-effects regression for <3.0 mmol/mol

| Variable | Coefficient | Std. Error | z | p-value | 95% CI Lower | 95% CI Upper |
|---|---|---|---|---|---|---|
| Moment 2 | 0.2201859 | 0.4368063 | 0.5 | 0.614 | -0.6359387 | 1.07631 |
| Moment 3 | 0.1413503 | 0.4692446 | 0.3 | 0.763 | -0.7783523 | 1.061053 |
| Moment 4 | 0.3477531 | 0.5116773 | 0.68 | 0.497 | -0.6551159 | 1.350622 |
| Moment 5 | 0.2862164 | 0.5663762 | 0.51 | 0.613 | -0.8238606 | 1.396293 |
| Treatment arm (1=treatment) | -0.8959071 | 0.9068855 | -0.99 | 0.323 | -2.67337 | 0.8815559 |
| Sex (1=male) | -0.9889071 | 0.7280655 | -1.36 | 0.174 | -2.415889 | 0.4380751 |
| Diabetic retinopathy | 1.687835 | 0.9561957 | 1.77 | 0.078 | -0.1862745 | 3.561944 |
| Duration of diabetes | -0.0154833 | 0.0324088 | -0.48 | 0.633 | -0.0790034 | 0.0480368 |
| Treatment arm × Moment | 0.0743553 | 0.1928122 | 0.39 | 0.7 | -0.3035497 | 0.4522603 |
| Constant | 3.544104 | 0.9379649 | 3.78 | 0.0 | 1.705727 | 5.382482 |

Per-protocol mixed-effects regression for TBR <3.0 mmol/mol

| Variable | Coefficient | Std. Error | z | p-value | 95% CI Lower | 95% CI Upper |
|---|---|---|---|---|---|---|
| Moment 2 | -0.1213903 | 0.1541844 | -0.79 | 0.431 | -0.4235863 | 0.1808056 |
| Moment 3 | -0.0541982 | 0.1655696 | -0.33 | 0.743 | -0.3787087 | 0.2703124 |
| Moment 4 | -0.0826001 | 0.1806199 | -0.46 | 0.647 | -0.4366086 | 0.2714085 |
| Moment 5 | 0.0556049 | 0.1999268 | 0.28 | 0.781 | -0.3362445 | 0.4474542 |
| Treatment arm (1=treatment) | -0.2148501 | 0.2609268 | -0.82 | 0.41 | -0.7262572 | 0.2965571 |
| Sex (1=male) | -0.2408729 | 0.1706193 | -1.41 | 0.158 | -0.5752806 | 0.0935348 |
| Diabetic retinopathy | 0.6466465 | 0.2238095 | 2.89 | 0.004 | 0.207988 | 1.085305 |
| Duration of diabetes | -0.0060507 | 0.0075862 | -0.8 | 0.425 | -0.0209194 | 0.0088179 |
| Treatment arm × Moment | -0.0139882 | 0.0679968 | -0.21 | 0.837 | -0.1472596 | 0.1192831 |
| Constant | 0.6535042 | 0.2359413 | 2.77 | 0.006 | 0.1910678 | 1.115941 |

Per-protocol mixed-effects regression for Mean Glucose (mmol/mol)

| Variable | Coefficient | Std. Error | z | p-value | 95% CI Lower | 95% CI Upper |
|---|---|---|---|---|---|---|

| | | | | | | |
|---|---|---|---|---|---|---|
| Moment 2 | -3.795148 | 3.694181 | -1.03 | 0.304 | -11.03561 | 3.445314 |
| Moment 3 | -3.228711 | 3.969464 | -0.81 | 0.416 | -11.00872 | 4.551296 |
| Moment 4 | 0.1716486 | 4.327268 | 0.04 | 0.968 | -8.309641 | 8.652938 |
| Moment 5 | -1.108398 | 4.789884 | -0.23 | 0.817 | -10.4964 | 8.279602 |
| Treatment arm (1=treatment) | 10.18696 | 11.90388 | 0.86 | 0.392 | -13.14422 | 33.51814 |
| Sex (1=male) | 18.7868 | 11.26646 | 1.67 | 0.095 | -3.295051 | 40.86866 |
| Diabetic retinopathy | -24.88933 | 14.8087 | -1.68 | 0.093 | -53.91384 | 4.135178 |
| Duration of diabetes | 1.008354 | 0.5018837 | 2.01 | 0.045 | 0.0246796 | 1.992028 |
| Treatment arm × Moment | -2.631012 | 1.631633 | -1.61 | 0.107 | -5.828954 | 0.5669296 |
| Constant | 153.8412 | 13.89288 | 11.07 | 0.0 | 126.6117 | 181.0708 |

Per-protocol mixed-effects regression for GMI

| Variable | Coefficient | Std. Error | z | p-value | 95% CI Lower | 95% CI Upper |
|---|---|---|---|---|---|---|
| Moment 2 | -0.0923574 | 0.0874495 | -1.06 | 0.291 | -0.2637553 | 0.0790405 |
| Moment 3 | -0.0814213 | 0.0939661 | -0.87 | 0.386 | -0.2655916 | 0.1027489 |
| Moment 4 | -0.0021894 | 0.1024361 | -0.02 | 0.983 | -0.2029605 | 0.1985816 |
| Moment 5 | -0.027584 | 0.1133873 | -0.24 | 0.808 | -0.2498189 | 0.194651 |
| Treatment arm (1=treatment) | 0.2519624 | 0.2824227 | 0.89 | 0.372 | -0.3015759 | 0.8055007 |
| Sex (1=male) | 0.4474179 | 0.2674184 | 1.67 | 0.094 | -0.0767125 | 0.9715483 |
| Diabetic retinopathy | -0.5917273 | 0.3514969 | -1.68 | 0.092 | -1.280649 | 0.097194 |
| Duration of diabetes | 0.0240904 | 0.0119126 | 2.02 | 0.043 | 0.000742 | 0.0474387 |
| Treatment arm × Moment | -0.0630559 | 0.0386245 | -1.63 | 0.103 | -0.1387584 | 0.0126467 |
| Constant | 6.98937 | 0.3297242 | 21.2 | 0.0 | 6.343123 | 7.635618 |

Per-protocol mixed-effects regression for Coefficient of Variation (CV)

| Variable | Coefficient | Std. Error | z | p-value | 95% CI Lower | 95% CI Upper |
|---|---|---|---|---|---|---|
| Moment 2 | 0.1512727 | 0.8054653 | 0.19 | 0.851 | -1.42741 | 1.729956 |
| Moment 3 | 1.372535 | 0.865402 | 1.59 | 0.113 | -0.3236222 | 3.068691 |

| | | | | | | |
|---|---|---|---|---|---|---|
| Moment 4 | 0.9857304 | 0.9435118 | 1.04 | 0.296 | -0.8635188 | 2.83498 |
| Moment 5 | 1.697771 | 1.044378 | 1.63 | 0.104 | -0.3491715 | 3.744714 |
| Treatment arm (1=treatment) | 0.6187198 | 1.999488 | 0.31 | 0.757 | -3.300204 | 4.537644 |
| Sex (1=male) | -1.534381 | 1.75865 | -0.87 | 0.383 | -4.981271 | 1.912509 |
| Diabetic retinopathy | 2.154741 | 2.310785 | 0.93 | 0.351 | -2.374314 | 6.683797 |
| Duration of diabetes | 0.0808509 | 0.0783176 | 1.03 | 0.302 | -0.0726488 | 0.2343507 |
| Treatment arm × Moment | -0.5420861 | 0.3556662 | -1.52 | 0.127 | -1.239179 | 0.1550067 |
| Constant | 34.76011 | 2.208412 | 15.74 | 0.0 | 30.43171 | 39.08852 |

Per-protocol mixed-effects regression for Standard Deviation (SD)( mmol/mol)

| Variable | Coefficient | Std. Error | z | p-value | 95% CI Lower | 95% CI Upper |
|---|---|---|---|---|---|---|
| Moment 2 | -0.4421176 | 1.831465 | -0.24 | 0.809 | -4.031722 | 3.147487 |
| Moment 3 | 2.064917 | 1.967867 | 1.05 | 0.294 | -1.792032 | 5.921865 |
| Moment 4 | 2.242313 | 2.14534 | 1.05 | 0.296 | -1.962475 | 6.447101 |
| Moment 5 | 2.772685 | 2.37469 | 1.17 | 0.243 | -1.881621 | 7.426992 |
| Treatment arm (1=treatment) | 4.34766 | 5.203974 | 0.84 | 0.403 | -5.851941 | 14.54726 |
| Sex (1=male) | 4.530914 | 4.782988 | 0.95 | 0.343 | -4.843571 | 13.9054 |
| Diabetic retinopathy | -5.578506 | 6.285945 | -0.89 | 0.375 | -17.89873 | 6.741719 |
| Duration of diabetes | 0.5167932 | 0.2130406 | 2.43 | 0.015 | 0.0992413 | 0.9343451 |
| **Treatment arm × Moment** | **-1.850285** | **0.8088363** | **-2.29** | **0.022** | -3.435575 | -0.2649954 |
| Constant | 52.29096 | 5.939596 | 8.8 | 0.0 | 40.64956 | 63.93235 |

Per-protocol mixed-effects regression for Number of Hypoglycemia Events

| Variable | Coefficient | Std. Error | z | p-value | 95% CI Lower | 95% CI Upper |
|---|---|---|---|---|---|---|

| | | | | | | |
|---|---|---|---|---|---|---|
| Moment 2 | 0.2165929 | 0.8035134 | 0.27 | 0.788 | -1.358264 | 1.79145 |
| Moment 3 | 0.2858406 | 0.8633313 | 0.33 | 0.741 | -1.406258 | 1.977939 |
| Moment 4 | 0.5181365 | 0.941222 | 0.55 | 0.582 | -1.326625 | 2.362898 |
| Moment 5 | 0.8492282 | 1.041844 | 0.82 | 0.415 | -1.192748 | 2.891205 |
| Treatment arm (1=treatment) | -1.70297 | 2.121185 | -0.8 | 0.422 | -5.860416 | 2.454476 |
| Sex (1=male) | -2.311902 | 1.907171 | -1.21 | 0.225 | -6.049889 | 1.426085 |
| Diabetic retinopathy | 3.733581 | 2.506202 | 1.49 | 0.136 | -1.178484 | 8.645646 |
| Duration of diabetes | -0.0569427 | 0.0849399 | -0.67 | 0.503 | -0.223422 | 0.1095365 |
| Treatment arm × Moment | 0.1343619 | 0.3548318 | 0.38 | 0.705 | -0.5610956 | 0.8298193 |
| Constant | 9.449818 | 2.381382 | 3.97 | 0.0 | 4.782396 | 14.11724 |

Per-protocol mixed-effects regression for Hypoglycemia Duration

| Variable | Coefficient | Std. Error | z | p-value | 95% CI Lower | 95% CI Upper |
|---|---|---|---|---|---|---|
| Moment 2 | 10.76201 | 8.910666 | 1.21 | 0.227 | -6.702571 | 28.2266 |
| Moment 3 | 18.2818 | 9.570167 | 1.91 | 0.056 | -0.4753864 | 37.03898 |
| Moment 4 | 19.45721 | 10.43826 | 1.86 | 0.062 | -1.001414 | 39.91582 |
| Moment 5 | 10.1524 | 11.55407 | 0.88 | 0.38 | -12.49316 | 32.79796 |
| Treatment arm (1=treatment) | -1.687113 | 15.97549 | -0.11 | 0.916 | -32.9985 | 29.62428 |
| Sex (1=male) | -17.04131 | 11.27427 | -1.51 | 0.131 | -39.13846 | 5.05585 |
| Diabetic retinopathy | 20.46163 | 14.796 | 1.38 | 0.167 | -8.537992 | 49.46124 |
| Duration of diabetes | 0.3092253 | 0.5015109 | 0.62 | 0.538 | -0.6737181 | 1.292169 |
| Treatment arm × Moment | -1.300976 | 3.931114 | -0.33 | 0.741 | -9.005817 | 6.403865 |
| Constant | 71.02401 | 15.15117 | 4.69 | 0.0 | 41.32827 | 100.7198 |

# Electronic supplementary material Table 4

**Supplementary Table. ANCOVA model for Perceived Hypoglycemia (after multiple imputation, N=41)**

| Variable | Coefficient | Std. Error | t | p-value | 95% CI |
|---|---|---|---|---|---|
| Perceived hypoglycemia at baseline (HFS) | 0.031 | 0.149 | 0.21 | 0.836 | -0.272 to 0.335 |
| **Treatment group (ref=control)** | **-0.376** | **0.689** | **-0.55** | **0.589** | **-1.781 to 1.028** |
| Diabetes duration (years) | -0.041 | 0.032 | -1.28 | 0.212 | -0.106 to 0.025 |
| Diabetic retinopathy (yes vs no) | 0.748 | 0.935 | 0.80 | 0.430 | -1.163 to 2.659 |
| Sex (female vs male) | 0.622 | 0.713 | 0.87 | 0.389 | -0.830 to 2.074 |
| Constant | 1.031 | 0.894 | 1.15 | 0.257 | -0.790 to 2.851 |

# Electronic supplementary material Table 5

**Supplementary Tables. ANCOVA models after multiple imputation (N=41)**

Table 5.1. ANCOVA model for Hypoglycemia Fear Survey – Worry subscale

| Variable | Coefficient | Std. Error | t | p-value | 95% CI |
|---|---|---|---|---|---|
| Worry (baseline, HFS) | 0.795 | 0.139 | 5.73 | <0.001 | 0.511 to 1.079 |
| Treatment group (ref=control) | -3.906 | 3.794 | -1.03 | 0.311 | -11.647 to 3.835 |
| Diabetes duration (years) | 0.106 | 0.185 | 0.57 | 0.573 | -0.279 to 0.490 |
| Diabetic retinopathy (yes vs no) | -3.288 | 5.330 | -0.62 | 0.543 | -14.239 to 7.663 |
| Sex (female vs male) | 3.400 | 3.940 | 0.86 | 0.395 | -4.632 to 11.433 |
| Constant | 8.719 | 7.310 | 1.19 | 0.242 | -6.198 to 23.637 |

Table 5.2. ANCOVA model for Hypoglycemia Fear Survey – Avoidance subscale

| Variable | Coefficient | Std. Error | t | p-value | 95% CI |
|---|---|---|---|---|---|
| Avoidance (baseline, HFS) | 0.301 | 0.159 | 1.89 | 0.068 | -0.023 to 0.624 |
| **Treatment group (ref=control)** | **-2.442** | **0.986** | **-2.48** | **0.019** | **-4.452 to -0.432** |
| Diabetes duration (years) | -0.008 | 0.048 | -0.16 | 0.876 | -0.106 to 0.091 |
| Diabetic retinopathy (yes vs no) | -1.122 | 1.308 | -0.86 | 0.398 | -3.791 to 1.548 |
| Sex (female vs male) | 1.440 | 0.979 | 1.47 | 0.151 | -0.553 to 3.432 |
| Constant | 11.326 | 2.425 | 4.67 | <0.001 | 6.387 to 16.264 |

Table 5.3. ANCOVA model for Hypoglycemia Fear Survey – Hyperglycemia subscale

| Variable | Coefficient | Std. Error | t | p-value | 95% CI |
|---|---|---|---|---|---|

| | | | | | |
|---|---|---|---|---|---|
| Hyperglycemia (baseline, HFS) | 0.597 | 0.171 | 3.50 | 0.001 | 0.249 to 0.945 |
| Treatment group (ref=control) | 0.212 | 0.970 | 0.22 | 0.828 | -1.768 to 2.192 |
| Diabetes duration (years) | -0.030 | 0.044 | -0.69 | 0.497 | -0.120 to 0.060 |
| Diabetic retinopathy (yes vs no) | 0.369 | 1.300 | 0.28 | 0.778 | -2.289 to 3.027 |
| Sex (female vs male) | 0.886 | 1.043 | 0.85 | 0.402 | -1.239 to 3.011 |
| Constant | 2.298 | 1.621 | 1.42 | 0.166 | -1.006 to 5.602 |

# Electronic supplementary material Table 6

## ANCOVA (no imputation): HbA1c at follow-up (adjusted for baseline)

*Number of observations: 39*
*F(5, 33) = 54.58; Prob > F = 0.0000*
*R-squared = 0.8536; Root MSE = 0.48573*

| Variable | Coefficient | Robust SE | t | p-value | 95% CI Lower | 95% CI Upper |
|---|---|---|---|---|---|---|
| Treatment arm (1=treatment) | -0.3135188 | 0.1602085 | -1.96 | 0.059 | -0.6394655 | 0.0124279 |
| Baseline HbA1c | 0.9435759 | 0.0835291 | 11.3 | 0.0 | 0.7736347 | 1.113517 |
| Sex (1=male) | -0.2146592 | 0.1959682 | -1.1 | 0.281 | -0.6133595 | 0.1840412 |
| Duration of diabetes | 0.0076664 | 0.0063463 | 1.21 | 0.236 | -0.0052451 | 0.020578 |
| Diabetic retinopathy | -0.1004726 | 0.2226208 | -0.45 | 0.655 | -0.553398 | 0.3524529 |
| Constant | 0.5705791 | 0.6251876 | 0.91 | 0.368 | -0.7013746 | 1.842533 |

## ANCOVA (no imputation): Hypoglycemia awareness at follow-up (adjusted for baseline)

*Number of observations: 39*
*F(5, 33) = 0.60; Prob > F = 0.6990*
*R-squared = 0.0757; Root MSE = 2.0968*

| Variable | Coefficient | Robust SE | t | p-value | 95% CI Lower | 95% CI Upper |
|---|---|---|---|---|---|---|
| Treatment arm (1=treatment) | -0.3421023 | 0.6616936 | -0.52 | 0.609 | -1.688328 | 1.004124 |
| Baseline hypoglycemia awareness | 0.0332891 | 0.1041802 | 0.32 | 0.751 | -0.1786671 | 0.2452453 |
| Sex (1=male) | 0.5969485 | 0.7193715 | 0.83 | 0.413 | -0.8666239 | 2.060521 |
| Duration of diabetes | -0.038862 | 0.0346179 | -1.12 | 0.27 | -0.1092927 | 0.0315686 |
| Diabetic retinopathy | 0.6830249 | 0.9085739 | 0.75 | 0.458 | -1.165483 | 2.531532 |
| Constant | 1.007842 | 0.7964181 | 1.27 | 0.215 | -0.6124828 | 2.628167 |

ANCOVA (no imputation): Hypoglycemia Fear (EsHFS) – Worry subscale at follow-up (adjusted for baseline)

*Number of observations: 39*

*F(5, 33) = 12.55; Prob > F = 0.0000*

*R-squared = 0.6178; Root MSE = 10.358*

| Variable | Coefficient | Robust SE | t | p-value | 95% CI Lower | 95% CI Upper |
|---|---|---|---|---|---|---|
| Treatment arm (1=treatment) | -3.556953 | 3.50934 | -1.01 | 0.318 | -10.69676 | 3.582852 |
| Baseline EsHFS – Worry | 0.8475689 | 0.1184632 | 7.15 | 0.0 | 0.6065536 | 1.088584 |
| Sex (1=male) | 2.802468 | 3.602846 | 0.78 | 0.442 | -4.527577 | 10.13251 |
| Duration of diabetes | 0.0677962 | 0.1511309 | 0.45 | 0.657 | -0.2396818 | 0.3752743 |
| Diabetic retinopathy | -3.989681 | 4.296402 | -0.93 | 0.36 | -12.73078 | 4.751415 |
| Constant | 7.01737 | 5.847393 | 1.2 | 0.239 | -4.879239 | 18.91398 |

ANCOVA (no imputation): Hypoglycemia Fear (EsHFS) – Avoidance subscale at follow-up (adjusted for baseline)

*Number of observations: 39*

*F(5, 33) = 6.15; Prob > F = 0.0004*

*R-squared = 0.3523; Root MSE = 2.8739*

| Variable | Coefficient | Robust SE | t | p-value | 95% CI Lower | 95% CI Upper |
|---|---|---|---|---|---|---|
| Treatment arm (1=treatment) | -2.388465 | 1.188922 | -2.01 | 0.053 | -4.807345 | 0.0304144 |
| Baseline EsHFS – Avoidance | 0.3054942 | 0.1978771 | 1.54 | 0.132 | -0.0970899 | 0.7080783 |
| Sex (1=male) | 1.405648 | 0.9946373 | 1.41 | 0.167 | -0.6179565 | 3.429253 |
| Duration of diabetes | -0.0060038 | 0.0602125 | -0.1 | 0.921 | -0.128507 | 0.1164994 |
| Diabetic retinopathy | -1.197683 | 1.094568 | -1.09 | 0.282 | -3.424598 | 1.029232 |
| Constant | 11.2332 | 2.982685 | 3.77 | 0.001 | 5.164885 | 17.30152 |

ANCOVA (no imputation): Hyperglycemia (EsHFS) at follow-up (adjusted for baseline)

*Number of observations: 38*

*F(5, 32) = 4.64; Prob > F = 0.0027*

*R-squared = 0.3500; Root MSE = 2.9046*

| Variable | Coefficient | Robust SE | t | p-value | 95% CI Lower | 95% CI Upper |
|---|---|---|---|---|---|---|
| Treatment arm (1=treatment) | 0.2768578 | 1.01872 | 0.27 | 0.788 | -1.798208 | 2.351923 |
| Baseline EsHFS – Hyperglycemia | 0.5897673 | 0.1505778 | 3.92 | 0.0 | 0.2830503 | 0.8964842 |
| Sex (1=male) | 0.8537253 | 0.9884497 | 0.86 | 0.394 | -1.159681 | 2.867131 |
| Duration of diabetes | -0.0273939 | 0.0437937 | -0.63 | 0.536 | -0.1165987 | 0.061811 |
| Diabetic retinopathy | 0.2562211 | 1.17633 | 0.22 | 0.829 | -2.139884 | 2.652326 |
| Constant | 2.313961 | 1.232703 | 1.88 | 0.07 | -0.1969725 | 4.824894 |
